# A new reference implementation of the PSICQUIC web service

**DOI:** 10.1093/nar/gkt392

**Published:** 2013-05-11

**Authors:** Noemi del-Toro, Marine Dumousseau, Sandra Orchard, Rafael C. Jimenez, Eugenia Galeota, Guillaume Launay, Johannes Goll, Karin Breuer, Keiichiro Ono, Lukasz Salwinski, Henning Hermjakob

**Affiliations:** ^1^EMBL–European Bioinformatics Institute, Hinxton, Cambridge CB10 1SD, UK, ^2^Department of Biology, University of Rome Tor Vergata, Rome, 00133, Italy, ^3^Institut de Biologie et Chimie des Protéines, UMR5086 CNRS/Lyon1 University, 69367, France, ^4^The J. Craig Venter Institute, Rockville, Maryland, MD 20850, USA, ^5^Department of Molecular Biology and Biochemistry—Simon Fraser University, Burnaby, British Columbia V5A 1S6, Canada, ^6^Department of Medicine, University of California, San Diego, La Jolla, CA 92093, USA, ^7^Department of Bioengineering, University of California, San Diego, La Jolla, CA 92093, USA and ^8^Department of Energy Institute for Genomics and Proteomics, University of California, Los Angeles, Los Angeles, CA 90095, USA

## Abstract

The Proteomics Standard Initiative Common QUery InterfaCe (PSICQUIC) specification was created by the Human Proteome Organization Proteomics Standards Initiative (HUPO-PSI) to enable computational access to molecular-interaction data resources by means of a standard Web Service and query language. Currently providing >150 million binary interaction evidences from 28 servers globally, the PSICQUIC interface allows the concurrent search of multiple molecular-interaction information resources using a single query. Here, we present an extension of the PSICQUIC specification (version 1.3), which has been released to be compliant with the enhanced standards in molecular interactions. The new release also includes a new reference implementation of the PSICQUIC server available to the data providers. It offers augmented web service capabilities and improves the user experience. PSICQUIC has been running for almost 5 years, with a user base growing from only 4 data providers to 28 (April 2013) allowing access to 151 310 109 binary interactions. The power of this web service is shown in PSICQUIC View web application, an example of how to simultaneously query, browse and download results from the different PSICQUIC servers. This application is free and open to all users with no login requirement (http://www.ebi.ac.uk/Tools/webservices/psicquic/view/main.xhtml).

## INTRODUCTION

One of the main issues currently facing the scientific community is the integration of data generated by the many different instruments and software platforms used in high-throughput experiments. The Human Proteome Organization Proteomics Standards Initiative (HUPO-PSI) was founded with the aim of developing standards to unify the diversity of data produced by proteomics experiments (1). In 2004, the Molecular Interaction (MI) group of the PSI jointly published a community-standard XML data model for the representation and exchange of protein-interaction data (2). The same work group subsequently published the Minimum Information about a Molecular Interaction Experiment (MIMIx) (3) guidelines, defining a list of parameters to be supplied when describing experimental molecular-interaction data in a journal publication. A number of public interaction databases have gone still further, forming the International Molecular Exchange consortium (IMEx) (4,5) to facilitate assembly of a single non-redundant set of consistently curated protein-interaction data. This original XML model was later further refined and in 2007 was supplemented by a simple tab delimited format, PSI-MITAB (6). These formats have been widely adopted by molecular-interaction databases (7), enabling the initial development of the Proteomics Standard Initiative Common QUery InterfaCe (PSICQUIC) service.

The PSICQUIC (8) specification is defined by means of a Web Service, with a clean-cut set of methods that have as input a query in MIQL (Molecular Interactions Query Language). The initial release of PSICQUIC supported only the very limited set of 15 fields of PSI-MITAB 2.5, which represented a simplistic description of molecular-interaction data. Following the development of extended PSI-MITAB formats (6) (version 2.6 and more recently 2.7), the number of fields has been increased to be fully compliant with MIMIx and enable presentation of IMEx-standard curated data (4). All these changes have resulted in the development of a new PSICQUIC specification encompassing extensions to the MIQL query language and a completely new implementation of the PSICQUIC reference server. Accompanying documentation helps data suppliers to easily migrate to the new, more efficient and feature-rich server.

## MATERIALS AND METHODS

### PSICQUIC specification

PSICQUIC defines a minimum set of standard SOAP and REST methods to be implemented by every molecular-interaction provider. These methods accept a MIQL query as input and return, as output, molecular-interaction information in one of the standard formats (PSI-XML 2.5, PSI-MITAB 2.5, PSI-MITAB 2.6, PSI-MITAB 2.7).

The PSICQUIC SOAP-based services are defined through a standard WSDL specification that all implementations must comply with. This definition has remained stable since PSICQUIC specification version 1.1; however, the capability to return the new PSI-MITAB versions has been added. Among the various methods described in the specification, the most flexible one is the getByQuery. It can be used to perform rather complex queries, as it accepts all the fields defined in MIQL. The results are returned, as specified by the user, in one of the standard formats (PSI-XML or PSI-MITAB). The remaining SOAP methods do not directly use MIQL. A summary of the main methods is shown in Table 1 and Table 2, and further information about their different options is available in the PSICQUIC specification for SOAP available in the PSICQUIC project web (http://code.google.com/p/psicquic/wiki/PsicquicSpec_1_3_Soap).

**Table 1.** Summary of the main available methods in SOAP

Method name	Description
getByQuery	Retrieve data using an MIQL query
getByInteractor	Retrieve data using a specific participant identifier (equivalent MIQL field identifier)
getByInteractorList	Retrieve data using a list of participant identifiers. This method can be used to retrieve interactions where the two or more participants passed as arguments are found. To do so, set the operand to AND.
getByInteraction	Retrieve a specific interaction using its identifier (equivalent MIQL field interaction_id)
getByInteractionList	Retrieve a list of interactions, using the identifiers

**Table 2.** SOAP methods to retrieve information about the service itself (metadata)

Method name	Description
getSupportedReturn Types	Returns the list of possible formats for the retrieved data
getVersion	Gets the version of the service
getProperties	Retrieve a list of the property objects defined in a service by the provider. Each property object have a key and a value
getProperty	Retrieve a property from the service

**Figure 1.**
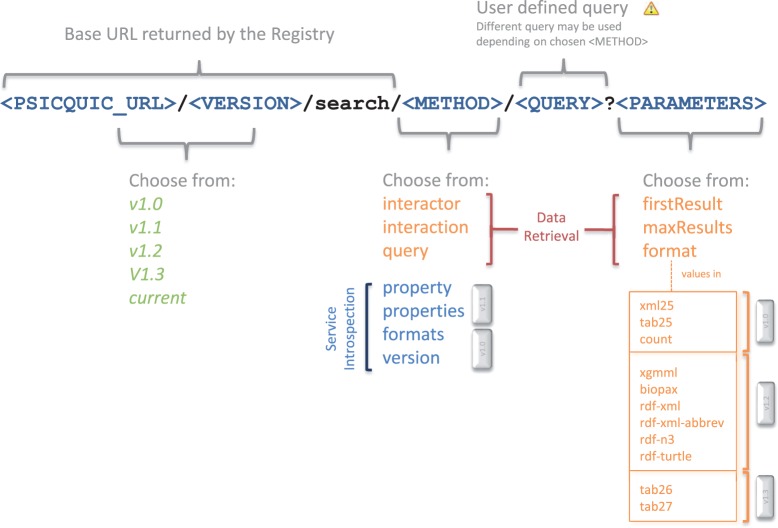
Structure of the URL to fetch data from PSICQUIC service.

In addition to the SOAP-based protocol, PSICQUIC also implements a set of RESTful services to make it possible to retrieve data over HTTP using simple URLs. This protocol can also be used to access molecular interactions through scripting languages and supports other common output formats such as Resource Description Framework (RDF), Biological Pathway Exchange (BioPAX) and eXtensible Graph Markup and Modeling Language (XGMML). It should be noticed that these new formats have existed since PSICQUIC specification version 1.2, and they are only available through the REST service. As in SOAP, there is an ample set of the methods to choose from. Figure 1 demonstrates how to access the information by means of HTTP GET requests. A template URL describes the main methods with the different options and the outputs. In the PSICQUIC specification for REST available in (http://code.google.com/p/psicquic/wiki/PsicquicSpec_1_3_Rest), a more extensive version of the methods and options is presented.

### MIQL

The main input to the PSICQUIC web service is a query written in the MIQL query language. MIQL defines a set of standard fields to query molecular-interaction data, extending the syntax of the Apache Lucene query language on which it is based.

MIQL has also been updated with the new data fields; these new fields allow users to filter (or query) molecular interactions with novel criteria that adjust the result to their needs and reduce post-processing steps. The retrieval of information that was previously unavailable has thus been enabled. For example, in the new PSI-MITAB 2.6, the ‘complex expansion’ field is introduced. Thanks to this field, and with the new PSICQUIC service, the user now is able to distinguish the results that come from a original binary interaction or a binary pair resulting from the expansion of a n-ary interaction with one of the expansion methods available (spoke expansion, bipartite expansion or matrix expansion); distinguishing this information was previously impossible. Adding the ‘stoichiometry’ field (included in PSI-MITAB 2.7) allows the retrieval of information about intra-molecular interactions and with the inclusion of the ‘features’ field, PSICQUIC is able to provide, for the first time, fully compliant MIMIx information.

In the updated PSI-MITAB formats, some information has been reallocated to the new columns. This removed some previously existing inconsistencies and made the records more accurate and easier to access through a MIQL query. See PSICQUIC extension for MIQL (http://code.google.com/p/psicquic/wiki/MiqlReference27) for a detailed description of the additional fields.

### Reference implementation

The open-source reference implementation described in this article has been wholly rewritten independently from the original (3) but remains backwards compatible with the previous versions of all the protocols. This new PSICQUIC service is based on Apache Solr indexing software (http://lucene.apache.org/solr/), which is a web application built on top of Apache Lucene technology. From the data provider perspective, the new open-source reference implementation of PSICQUIC allows a local PSICQUIC server to be easily set up and loaded with data provided as a valid PSI-MITAB file. It supports the original 15-column PSI-MITAB 2.5 as well as newer, PSI-MITAB 2.6 and PSI-MITAB 2.7 formats, with 36 and 42 columns respectively (see Table 3).

**Table 3.** Evolution of PSI-MITAB format

PSI-MITAB 2.5 (15 cols)	PSI-MITAB 2.6 (+21 cols)	PSI-MITAB 2.7 (+6 cols)
ID(s) interactor A & B	Experimental role(s) A &B	Features A & B
Alt. ID(s) interactor A & B	Biological role(s) A & B	Stoichiometry A & B
Alias(es) interactor A & B	Properties (CrossReference) A & B	Participant detection method A & B
Interaction detection method(s)	Type(s) of interactors A & B	
Publication 1st author(s)	Host organism	
Publication Identifier(s)	Expansion method(s)	
Taxid interactor A & B	Annotations A & B	
Interaction type(s)	Parameters	
Source database(s)	Creation/update date	
Interaction identifier(s)	Checksums A, B & interaction	
Confidence value(s)	Negative	

In addition to introducing support for the new PSI-MITAB versions and the MIQL extension, extensive restructuring of the code resulted in improved response time of the server. It also removed the restriction on the number of interactions that can be exported in the XGMML format used in Cytoscape (9,10), which previously existed in the REST protocol (sending small chunks of interactions until the file is completely transmitted instead of truncating it as it was before). All these improvements enhance the web service and the concurrent search of multiple molecular-interaction databases independently of the different clients.

### Server deployment

**Figure 2.**
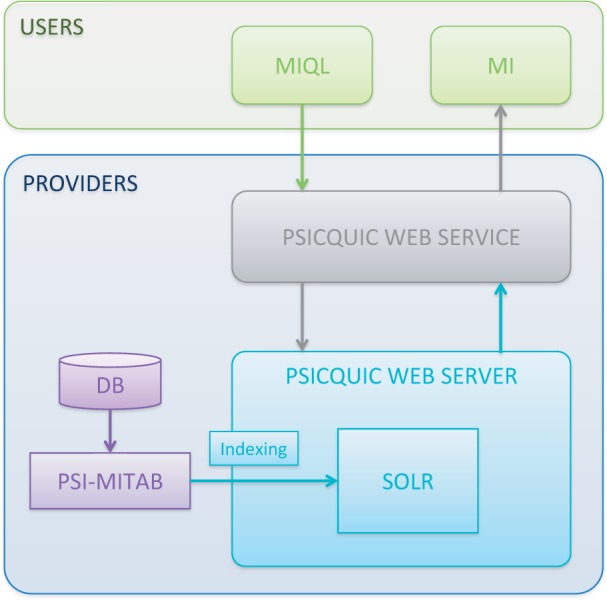
Dataflow followed by the reference implementation from its origin in the molecular-interaction databases to the end user through PSICQUIC.

The reference implementation source code can be downloaded from the PSICQUIC Google project repository (svn co http://psicquic.googlecode.com/svn/tags/psicquic-solr-ws-1.3.8). It includes a JAVA class to create the index from the PSI-MITAB file and a script that can be used to easily start the indexing process (bash indexMitab.sh /path/to/mitab-file solr-index-directory). The solr-index directory will contain the index, solr configuration files, solr schema and the solr.war file mandatory to run the solr application. More detailed information and other options to deploy a PSICQUIC server is available on the PSICQUIC website (https://code.google.com/p/psicquic/wiki/HowToInstallPsicquicSolr). In Figure 2 different elements required to build a PSICQUIC service from an interaction database are shown for clarification of this process. Solr indexing will enable the development of facilities such as visualization of statistical data through faceting, indexing from PSI-XML and data sorting. In addition to using the default implementation presented, providers can also implement their own systems to publish interactions as long as they meet the PSICQUIC specifications (http://code.google.com/p/psicquic/wiki/PsicquicSpecification).

### PSICQUIC clients

**Figure 3.**
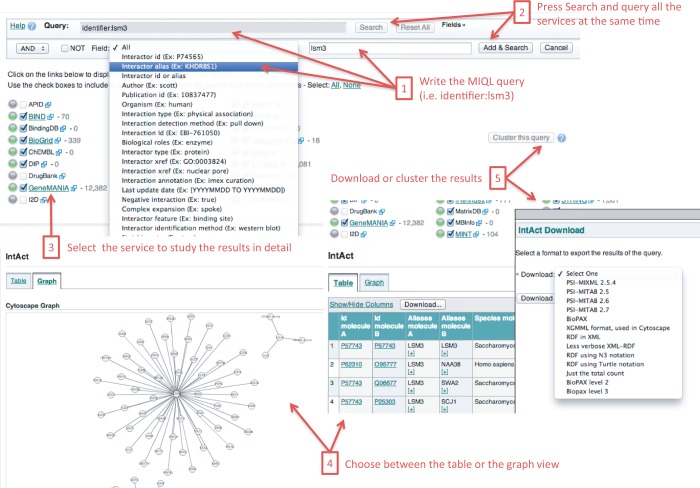
PSICQUIC View is a client for PSICQUIC services in which by formulating only one query fetches all the relevant molecular interactions available in the registered services. After the search, the user can choose from studying the results in more detail, viewing the interaction network, downloading or clustering the results.

In addition to using the services directly from the browser (in the case of REST) or, alternatively, create a custom client, there are several applications at users’ disposal for querying the web services programmatically. The PSICQUIC project site (http://psicquic.googlecode.com) offers open-source libraries for working with the different standards, JAVA clients to access the web services (http://code.google.com/p/psicquic/wiki/JavaClient), code examples for accessing from Perl (http://code.google.com/p/psicquic/wiki/PerlCodeSamples) and other scripts in Python (http://code.google.com/p/psicquic/wiki/PythonCodeSamples) and help for broad use cases. Important clients include the molecular interactions cluster (http://code.google.com/p/micluster), the PSICQUIC Client Plugin for Cytoscape (http://apps.cytoscape.org/apps/psicquicuniversalclient) or the PSICQUIC View (http://www.ebi.ac.uk/Tools/webservices/psicquic/view/main.xhtml). See Figure 3.

### PSICQUIC registry

**Figure 4.**
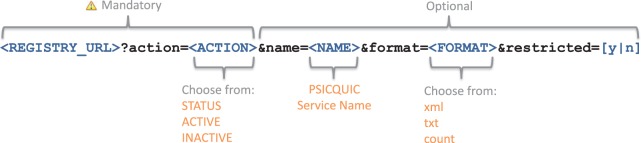
Structure of the URL to fetch data from PSICQUIC registry.

Users are expected to obtain the PSICQUIC web service SOAP or REST URLs by means of querying the PSICQUIC Registry. In addition to providing the necessary URLs, the registry is itself a REST web service, offering data on the number of interactions per service, the status of each service, a statement as to whether the data are restricted or not, the version of the software used and a small description of the type of service given by means of tags. The PSICQUIC registry is currently hosted at the European Bioinformatics Institute (http://www.ebi.ac.uk/Tools/webservices/psicquic/registry/registry?action=STATUS). Figure 4 explains how to retrieve the information from the registry through HTTP. More information about the PSICQUIC registry is available at the PSICUIC project site (http://code.google.com/p/psicquic/wiki/Registry).

## DISCUSSION

In <5 years since its original implementation, PSICQUIC has grown from 4 to 28 providers supplying >150 million interactions, with additional services preparing to join. With the new reference implementation, we open the door to the additional new features such as sorting by different criteria (for example, the confidence score of the interactions) or faceting to retrieve statistics. Longer-term plans include direct indexing of the PSI-XML data to allow processing of the molecular-interaction data described in the original PSI-XML files, thus avoiding the currently necessary, lossy conversions between PSI-XML and PSI-MITAB formats. This, in turn, will enable the querying and retrieval of n-ary interactions rather than only binary pairs.

## FUNDING

European Commission grant PSIMEx [FP7-HEALTH-2007-223411]; National Institutes of Health [R01GM071909 to L.S.]; National Heart, Lung, and Blood Institute Proteomics Center Award [HHSN268201000035C to R.J]; Genome BC through the Pathogenomics of Innate Immunity (PI2) project; Foundation for the National Institutes of Health and the Canadian Institutes of Health Research under the Grand Challenges in Global Health Research Initiative [Grand Challenges ID: 419 to K.B.]; AllerGen [12ASI1;12B&B2] (to K.B.). Funding for open access charge: European Commission [FP7-HEALTH-2007-223411].

*Conflict of interest statement*. None declared.
